# COVID-19 Safety: Perspectives from Dental Students

**DOI:** 10.3390/dj12080264

**Published:** 2024-08-20

**Authors:** Man Hung, Daniel Yevseyevich, Amir Mohajeri, Nicole Hablitzel, Sharon Su, Martin S. Lipsky

**Affiliations:** 1 College of Dental Medicine, Roseman University of Health Sciences, South Jordan, UT 84095, USA; 2 Division of Public Health, University of Utah, Salt Lake City, UT 84108, USA; 3 Veteran Affairs Salt Lake City Health Care, Salt Lake City, UT 84148, USA; 4 Huntsman Cancer Institute, Salt Lake City, UT 84112, USA; 5 Institute on Aging, Portland State University, Portland, OR 97207, USA

**Keywords:** COVID-19, dental education, healthcare, safety protocol, students

## Abstract

Background: The coronavirus (COVID-19) pandemic created challenges for healthcare providers, especially dental professionals in practices and universities. This study investigated dental students’ concerns about COVID-19 and whether institutional communication influenced pandemic-related stressors. Methods: An online survey designed to elicit dental students’ concerns about COVID-19 was distributed to all dental students enrolled at a private non-profit academic institution in the Western United States from 26 April 2021 to 25 August 2021. Descriptive statistics were used to characterize the respondents, and Chi-square test and z-test analyses were used to compare students’ experiences. Results: A total of 92 dental students answered the survey. The respondents’ ages ranged from 22 to 47 years. Slightly more than half (50.5%) were male and 93.3% were non-Hispanic or non-Latino. Over one-fourth of the students (27.5%) reported that they either often or always felt anxious that they might become infected with the COVID-19 virus, with 16.3% of the students expressing concern about contracting COVID-19 while on campus. There were no statistically significant differences in anxiety levels about contracting COVID-19 from clinic patients or while on campus between the group of students who perceived the institution communicated effectively and those who did not. However, students who felt that the dental school clearly communicated COVID-19 protocols were more likely to believe that students took protocols seriously and expressed less concern about contracting COVID-19 by touching dental school items. Conclusions: About one-quarter of dental students expressed high levels of anxiety about being infected with COVID-19. Clear communication about COVID-19 protocols reduced students’ fear of contracting COVID-19 from dental school items and strengthened their belief that other students followed protocols.

## 1. Introduction

Severe acute respiratory syndrome coronavirus 2 (SARS-CoV-2) is the virus responsible for causing coronavirus disease 2019 (COVID-19) [1]. SARS-Cov-2 first emerged in December 2019, and by March 2020, the World Health Organization declared COVID-19 a global pandemic [2]. The pandemic had a profound impact on healthcare systems worldwide, leading to changes in clinical service delivery to mitigate the risk of infection transmission. Healthcare facilities worldwide adopted enhanced infection control measures, including the use of personal protective equipment (PPE), rigorous sanitation protocols, and the restructuring of patient care practices to minimize contact. Despite these measures, the fear of contracting COVID-19 caused many patients to delay or avoid seeking care, exacerbating existing health issues and leading to a backlog of untreated conditions [3]. In dental care, the pandemic presented unique challenges due to the high risk of transmission associated with dental procedures. Dental treatments often involve close contact and the aerosolization of oral secretions, which can facilitate the spread of respiratory pathogens like SARS-CoV-2. As a result, dental practices adapted, by implementing new infection control protocols and limiting treatments to urgent and emergency care.

Similarly, the risk of COVID-19 transmission [4] prompted dental schools to modify their infection control protocols. Many dental schools adopted measures recommended by the Centers for Disease Control and Prevention (CDC) and the American Dental Association (ADA) to mitigate transmission risk [5]. These guidelines aimed to protect dental professionals and patients from exposure to SARS-CoV-2. Key measures included limiting dental treatments to emergency and urgent care only, effectively postponing elective and routine procedures [6]. This approach sought to reduce the number of aerosol-generating procedures performed, thereby decreasing the potential for viral transmission within dental clinics. Additionally, dental schools introduced enhanced PPE protocols, including N95 respirators, face shields, gowns, and gloves for all patient interactions. Beyond the clinical environment, dental schools also adjusted their educational methods to meet social distancing and public health guidelines. The transition to distance and online learning became an important tool to ensure the safety of students and faculty. The rapid change to online learning disrupted the usual educational routines and posed challenges in maintaining the quality and effectiveness of dental education.

Preclinical and clinical training also faced significant disruptions. Hands-on training and clinical rotations were either suspended or significantly reduced, and many institutions developed virtual simulations and remote learning modules to fill gaps left by the absence of in-person practice. This shift required students and faculty to adapt to new technologies and teaching methods, often with limited preparation time.

The sweeping changes to dental education brought on by the COVID-19 pandemic significantly increased anxiety and stress among students [7]. Dental students, who already deal with stressors such as rigorous coursework, clinical work, and patient management [8], faced additional challenges from the abrupt curricular changes. The transition to online learning, the closure of clinical facilities, and the implementation of new infection control protocols compounded the existing pressures on students [9]. Studies found that as dental schools reopened, students experienced apprehension regarding their institutions’ pandemic responses and newly implemented protocols, expressing concern about the impact of these changes on various aspects of their lives [10]. Academically, students worried about the disruption to their education, the effectiveness of online education for hands-on skills, and gaps in their practical training, all of which are crucial for their future professional competence [11]. The physical and mental health of dental students also emerged as a critical concern. The fear of contracting COVID-19 during clinical activities, combined with the stress of adapting to new safety protocols, contributed to increased anxiety levels [12]. The isolation brought on by social distancing further exacerbated feelings of stress and loneliness among students [13]. Additionally, the pandemic’s impact on the healthcare system and dental practices led to uncertainty about their future career prospects., Financially, the economic downturn affected many students and their families, leading to worries about tuition fees, student loans, and overall financial stability [5].

Despite widespread concerns about the impact of COVID-19 on dental education, there is a lack of research examining the relationship between dental students’ apprehensions and the clarity of their dental school’s communication regarding COVID-19 safety protocols. While the pandemic caused immediate and far-reaching effects, the unprecedented shifts in dental education continue to affect dental education [14,15,16]. Understanding student sentiments can help guide institutions in ensuring clear communication and maintaining morale, which have relevance for navigating current and future disruptions.

This study aimed to fill this gap by investigating dental students’ concerns about COVID-19 and whether institutional communication might influence pandemic-related stressors. Specifically, this study compared the responses of students who felt that their dental school communicated its COVID-19 safety protocols clearly with those who felt the school did not communicate effectively. By analyzing the differences between these two groups, the study sought to answer whether effective communication might mitigate anxiety and concerns among dental students during a public health crisis. The findings from this study can provide valuable insights for dental schools and other educational institutions about the role of effective communication, and help to develop strategies to support their students more effectively during ongoing and future crises.

## 2. Methods

### 2.1. Setting

This study took place at Roseman University of Health Sciences College of Dental Medicine (RUHS-CODM), a non-profit, private dental school in South Jordan, Utah. Utah is in the Western United States (US), and South Jordan is located in the south central area of Salt Lake City (SLC) County, a medium-sized metropolitan area of slightly more than 1 million people. From March 2020 to April 2020, 5.7% of the tested population at SLC area health centers tested positive for SARS-CoV-2, with lower incidence rates among younger individuals and higher hospitalization rates for the elderly [17]. In underresourced areas of the county, minorities accounted for more than half the positive cases [18].

Utah had a state vaccination rate slightly below the US average and an overall 2021 death rate for COVID-19 of 64.9 per 100,000, ranking in the bottom 5 states [19,20] with a state vaccination rate slightly below the US average. COVID-19 is disproportionately deadly for older age groups and minorities, and since Utah has the youngest median age of all US states and a lower percentage of racial/ethnic minorities compared to other states [21], a lower death rate would be expected in Utah based on demographics. During the study period, the ADA and the CDC published guidelines for healthcare settings [22,23]. RUHS-CODM used these guidelines to shape its COVID-19 policies.

### 2.2. Study Participants

Eligible study participants included the 400 RUHS-CODM students actively enrolled and in good academic standing during the study period of 26 April 2021 to 25 August, 2021. At the time of the survey, the students, at all levels, participated in direct patient care.

### 2.3. Survey Development and Administration

To develop the survey instrument, the authors conducted a comprehensive review of the literature about the impact of the COVID-19 pandemic on dental students. The goal was to create questions that would assess student perceptions about the contracting and spreading of COVID-19 and to evaluate the impact of communication on their perceptions.

The literature review identified several themes and concerns related to dental education and contracting and spreading infection during a public health crisis. Members of the RUHS-CODM faculty and students reviewed these key issues, and two research team members (MH and NH) used their input to draft questions related to COVID-19 and institutional communication effectiveness. To ensure face and content validity, additional faculty and students reviewed the draft survey and their feedback was incorporated into a revised version. The designers deliberately intended the questionnaire to be short to facilitate better response and completion rates.

The revised draft survey consisted of a section related to demographic information and another section about perceptions related to COVID-19 precautions, anxiety, concerns about contracting COVID, and institutional communication effectiveness. The draft survey was pilot-tested with four dental students to assess clarity, comprehensiveness, and the time required for completion. Their feedback was incorporated into the final survey to improve content validity and user-friendliness. The final survey consisted of twelve questions and took approximately five minutes to complete online.

The twelve questions included six demographic questions, five questions that focused on dental students’ experiences and perceptions about contracting or spreading COVID-19 in dental school (outlined in Table 1), and a question about whether the student felt the dental school clearly communicated its COVID-19 safety protocol.

### 2.4. Outcome Measures

The primary survey outcome measures were the levels of anxiety and concern among dental students regarding COVID-19 and the students’ perceptions of the dental school’s communication of COVID-19 protocols. Dependent variables included student anxiety about COVID-19 infection, concerns about contracting COVID-19 on campus, from patients, and from touching objects, and how seriously students felt their classmates took COVID-19 precautions. The independent variable was the perceived clarity of the dental school’s communication regarding COVID-19 safety protocols.

### 2.5. Statistical Analyses

Descriptive statistics were employed to characterize the survey respondents. For continuous variables, the range, mean, and 95% confidence interval (CI) were reported to provide a comprehensive summary of the data distribution and variability. Categorical variables were presented using frequency and proportion to illustrate the distribution of responses among the survey participants.

To compare the responses between students who believed that the communication of COVID-19 safety protocols was clear and those who did not, Chi-square tests and z-tests were utilized. These tests were chosen to assess the association and differences between the two groups. The statistical significance level was set at *p* < 0.05, indicating that differences with a *p*-value less than 0.05 were considered statistically significant. This threshold ensures that the findings have a high probability of not being due to random chance, thereby strengthening the validity of the results.

## 3. Results

Out of a total of 400 dental students, 92 (23%) participated in the survey. The respondent’s ages ranged from 22 to 47 years, with a mean age of 27.46 years (95% CI = 26.67–28.25 years; SD = 3.74 years). The gender distribution among the respondents was roughly equal. In the category of racial and ethnic demographics, individuals who identified as “White” constituted 68.9% of the sample. Table 2 provides a detailed description of the sample.

The data revealed a range of anxiety levels among dental students about contracting COVID-19, with 26.4% never feeling anxious, 20.9% feeling anxious often and 6.6% always feeling anxious. The opinions about their classmates’ adherence to COVID-19 precautions were mixed: 33.7% were neutral, 32.6% felt precautions were taken a little seriously, and only 2.2% believed they were taken extremely seriously. Concerns about contracting COVID-19 on campus were relatively low, with 46.7% expressing none, 37.0% little concern, and only 3.3% responded they were very much concerned.

Furthermore, 54.3% of dental students were not concerned about spreading COVID-19 through touching objects at dental school, whereas 32.6% were a little concerned. Similarly, concerns about contracting COVID-19 from patients in dental clinics show that 44.6% were not concerned at all, 38.0% were a little concerned, and only 3.3% were very much concerned. Overall, while the data suggest concern among dental students about COVID-19, the majority did not express high levels of anxiety or concern regarding infection from various sources.

Table 3 compared differences in the answers to questions related to COVID-19 between dental students who reported clear communication about COVID-19 precautions versus those who felt that communication was not clear. There was not a significant difference between the groups in terms of how often students felt anxious about being infected with COVID-19 (*p* = 0.799). However, perceptions of classmates’ adherence to precautions displayed a significant disparity (*p* = 0.006), with students who reported clear communication perceiving their peers as taking precautions more seriously. Concerns about contracting COVID-19 on campus showed a trend towards significance (*p* = 0.076), suggesting a possible influence of communication on these concerns. Significant differences emerged about the spread of COVID-19 from touching objects in the dental school environment (*p* = 0.043), where students with clear communication expressed less concern. Conversely, concerns about contracting the virus from patients in the dental clinic did not differ significantly between the groups (*p* = 0.680).

## 4. Discussion

This study examined dental students’ concerns about COVID-19 and investigated whether clear communication about safety protocols influenced student opinions. Previous research found that dental students experience higher levels of anxiety during the COVID-19 pandemic compared to the general population [9]. Our study supports these findings, revealing that approximately one-quarter of dental students expressed a high level of concern about becoming infected with COVID-19. This anxiety level is consistent with the findings of Akinbugbe and colleagues, who also identified heightened anxiety among dental students during the pandemic [24]. Somewhat surprisingly, communication clarity did not significantly affect the student’s perceived level of anxiety about contracting COVID-19.

In addition to exploring the prevalence of anxiety, our study made significant observations regarding the impact of communication. Among students who reported clear communication, about one-quarter believed their classmates took COVID-19 precautions somewhat or extremely seriously. This perception is concerning, since the oral cavity is a known reservoir for the SARS-CoV-2 virus and dental procedures can aerosolize oral secretions [25]. The potential for exposure to the coronavirus through aerosol-generating procedures led the Occupational Safety and Health Administration to classify dentists as a very high-risk category [26]. Dental students may be at greater risk because student clinics often occur in open spaces, procedures take longer, increasing exposure time, and trainees may be less adept at managing bio-aerosols.

Perhaps students believe their classmates fail to take COVID-19 precautions seriously because they observed peers breaking safety protocols. This perception aligns with research indicating that while students generally endorse the importance of infection control, they often struggle to comply with these recommendations in practice [27,28]. Observing non-compliance among peers can contribute to a general sense of laxity and skepticism about the overall seriousness of how colleagues take safety measures. In light of these observations, it becomes evident that effective communication alone may not be sufficient to ensure adherence to safety protocols. In addition to clear and consistent messaging about infection control measures, it may be important to implement robust systems for monitoring adherence to these protocols. Regular feedback on compliance, coupled with tangible consequences for non-compliance, can help reinforce the importance of adhering to safety measures. By providing continuous feedback, institutions can help students understand the real-world implications of their behavior and encourage a culture of accountability and responsibility. Moreover, institutions should consider creating supportive environments where students feel empowered to remind their peers about the importance of following protocols without fear of reprisal or social ostracism. Peer influence plays a significant role in behavior modification, and fostering an environment where students support each other in adhering to safety measures can lead to more consistent compliance.

A second finding was that effective communication significantly reduced the concern about the spread of COVID-19 from touching objects found in dental school. While not specifically measured, alleviating this concern might lower anxiety related to contracting COVID-19. Although the threat from COVID-19 is real, open communication with peers, tutors, and the educational team can foster trust and cooperation [29]. While this study examined whether the perceived effectiveness of communication affected concerns, it did not explore what constitutes effective communication. Key characteristics of effective communication include openness, accuracy, easy accessibility for all stakeholders, and real-time availability. For example, one strategy could be to publish a link on the school website or social media page [30,31] with answers to frequently asked questions that address concerns about changes to educational and clinical activities. This link could also provide a platform for students to ask questions and raise concerns anonymously.

### Limitations and Future Direction

This study has several limitations. First, the response rate was only 23%. However, such a response rate is high among studies without incentives. Our findings represent a single dental school and may not be widely generalized. Nonetheless, our results regarding anxiety about contracting COVID-19 match those of other studies. However, further research is needed to examine dental schools across the US and other countries to validate these findings.

Additionally, the burden of COVID-19 on dental students encompasses more than concerns about infection. Future investigations should consider the impact of effective communication on economic and social factors, the cancellation of scientific events, and strategies for optimizing communication. These areas are crucial for comprehensively understanding the pandemic’s effects on dental students and for developing effective communication about crisis management protocols.

## 5. Conclusions

This study found that approximately one-quarter of dental students at a dental school experienced high levels of anxiety about contracting COVID-19. Students who believed that the school’s COVID-19 protocols were communicated clearly, had fewer concerns about contracting the virus from touching objects and more confidence that their colleagues took safety protocols seriously. This suggests that effective communication of protocols might alleviate some students’ concerns. Further research is needed to develop strategies for optimizing crisis management and communication.

## Figures and Tables

**Table 1:** Questions on dental students’ experiences and perceptions about contracting or spreading COVID-19.

Questions	Response Options
Do dental students feel that their classmates are taking COVID-19 precautions seriously?	Not at all seriously, A little seriously, Neutral, Somewhat seriously, Extremely seriously
How often do dental students feel anxious that they might be infected with the COVID-19 virus?	Never, Occasionally, Sometimes, Often, Always
Are dental students concerned about contracting COVID-19 while on campus?	Not at all concerned, A little concerned, Concerned, Very much concerned, Extremely concerned
Are dental students concerned with contracting COVID-19 from the patients they see in the dental clinic?	Not at all concerned, A little concerned, Concerned, Very much concerned, Extremely concerned
Are dental students concerned about the spread of COVID-19 from touching objects found in dental school?	Not at all concerned, A little concerned, Concerned, Very much concerned, Extremely concerned

**Table 2:** Demographic characteristics (N = 92).

Variable	Min	Max	Mean (95% CI)	SD	*n* (%)
Age (in year)	22	47	27.46 (26.67–28.25)	3.74	
Gender					
Male					46 (50.5)
Female					45 (49.5)
Race					
Asian					27 (30)
Native Hawaiian or Other Pacific Islander					1 (1.1)
White					62 (68.9)
Ethnicity					
Hispanic or Latino					6 (6.7)
Non-Hispanic or Non-Latino					84 (93.3)
Marital status					
Married					33 (36.7)
Single					54 (60)
Other					3 (3.3)
Year in School					
Class of 2024 (first year)					46 (50)
Class of 2023 (second year)					21 (22.8)
Class of 2022 (third year)					18 (19.6)
Class of 2021 (fourth year)					7 (7.6)

**Table 3:** Group differences in concerns related to COVID-19.

Variable	With Clear Communication (*n* = 76)	Without Clear Communication (*n* = 16)	*p*-value
How often do dental students feel anxious that they might be infected with COVID-19?			
Never	19 (25.3)	5 (31.3)	0.799
Occasionally	19 (25.3)	4 (25)	
Sometimes	16 (21.3)	3 (18.8)	
Often	15 (20)	4 (25)	
Always	6 (8)	-	
Do dental students feel that their classmates are taking COVID-19 precautions seriously?			
Not at all seriously	5 (6.6)	5 (31.3)	0.006 *
A little seriously	22 (28.9)	8 (50)	
Neutral	30 (39.5)	1 (6.3)	
Somewhat seriously	17 (22.4)	2 (12.5)	
Extremely seriously	2 (2.6)	-	
Are dental students concerned about contracting COVID-19 while on campus?			
Not at all concerned	34 (44.7)	9 (56.3)	0.076
A little concerned	30 (39.5)	4 (25)	
Concerned	11 (14.5)	1 (6.3)	
Very much concerned	1 (1.3)	2 (12.5)	
Extremely concerned	-	-	
Are dental students concerned about the spread of COVID-19 from touching objects found in dental school?			
Not at all concerned	39 (51.3)	11 (68.8)	0.043 *
A little concerned	26 (34.2)	4 (25)	
Concerned	11 (14.5)	-	
Very much concerned	-	-	
Extremely concerned	-	1 (6.3)	
Are dental students concerned with contracting COVID-19 from the patients they see in the dental clinic?			
Not at all concerned	33 (43.4)	8 (50)	0.680
A little concerned	29 (38.2)	6 (37.5)	
Concerned	12 (15.8)	1 (6.3)	
Very much concerned	2 (2.6)	1 (6.3)	
Extremely concerned	-	-	

Note: * *p*-value < 0.05.

## Data Availability

Data may be available by obtaining IRB approval from Roseman University of Health Sciences and the corresponding author.
